# Epigallocatechin Gallate Affects the Structure of Chromatosomes, Nucleosomes and Their Complexes with PARP1

**DOI:** 10.3390/ijms241814187

**Published:** 2023-09-16

**Authors:** Tatiana V. Andreeva, Natalya V. Maluchenko, Anastasiya V. Efremenko, Alexander V. Lyubitelev, Anna N. Korovina, Dmitry A. Afonin, Mikhail P. Kirpichnikov, Vasily M. Studitsky, Alexey V. Feofanov

**Affiliations:** 1 Faculty of Biology, Lomonosov Moscow State University, 119234 Moscow, Russia; andreeva.tatyana.2014@post.bio.msu.ru (T.V.A.); maluchenko-msu@yandex.ru (N.V.M.); varanus-salvator@yandex.ru (A.V.L.); anna.korovina@gmail.com (A.N.K.); afoninmsu@outlook.com (D.A.A.); kirpichnikov@inbox.ru (M.P.K.); 2 Shemyakin-Ovchinnikov Institute of Bioorganic Chemistry, Russian Academy of Sciences, 117997 Moscow, Russia; aefr@mail.ru; 3 Fox Chase Cancer Center, Philadelphia, PA 19111-2497, USA

**Keywords:** epigallocatechin gallate, nucleosome, chromatosome, poly(ADP-ribose) polymerase 1, spFRET microscopy

## Abstract

The natural flavonoid epigallocatechin gallate has a wide range of biological activities, including being capable of binding to nucleic acids; however, the mechanisms of the interactions of epigallocatechin gallate with DNA organized in chromatin have not been systematically studied. In this work, the interactions of epigallocatechin gallate with chromatin in cells and with nucleosomes and chromatosomes in vitro were studied using fluorescent microscopy and single-particle Förster resonance energy transfer approaches, respectively. Epigallocatechin gallate effectively penetrates into the nuclei of living cells and binds to DNA there. The interaction of epigallocatechin gallate with nucleosomes in vitro induces a large-scale, reversible uncoiling of nucleosomal DNA that occurs without the dissociation of DNA or core histones at sub- and low-micromolar concentrations of epigallocatechin gallate. Epigallocatechin gallate does not reduce the catalytic activity of poly(ADP-ribose) polymerase 1, but causes the modulation of the structure of the enzyme–nucleosome complex. Epigallocatechin gallate significantly changes the structure of chromatosomes, but does not cause the dissociation of the linker histone. The reorganization of nucleosomes and chromatosomes through the use of epigallocatechin gallate could facilitate access to protein factors involved in DNA repair, replication and transcription to DNA and, thus, might contribute to the modulation of gene expression through the use of epigallocatechin gallate, which was reported earlier.

## 1. Introduction

**Figure 1:**
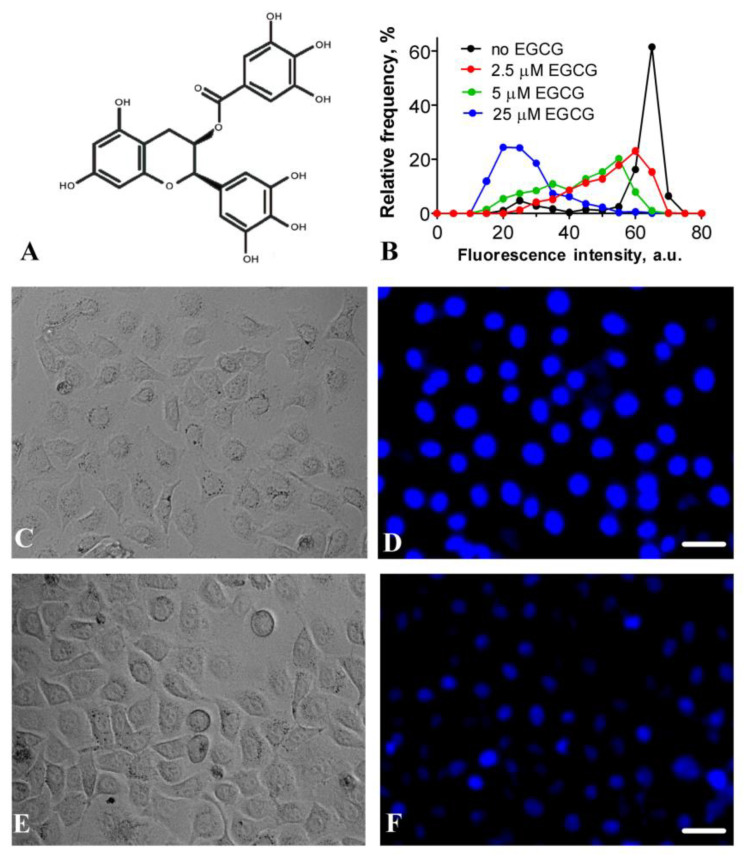
EGCG penetrates into the nuclei of living cells and competes for binding to DNA with Hoechst 33342. (**A**) Structure of EGCG. (**B**) Frequency distributions of cells by intensity of Hoechst 33342 fluorescence in the nuclei of HEK293 cells in the presence of different EGCG concentrations. Cells preincubated with 1 µM Hoechst 33342 for 3.5 h were incubated with indicated EGCG concentrations for 2.5 h in the complete medium. (**C**–**F**) Typical transmitted-light images of HEK293 cells (**C**,**E**) and fluorescent images of nuclei stained with Hoechst 33342 (**D**,**F**) in the absence (**D**) and presence (**F**) of 25 µM EGCG. Fluorescent images (**D**,**F**) were recorded under the same conditions; therefore, the signal intensities could be directly compared. Scale bar is 30 µm.

Epigallocatechin gallate (EGCG) is a natural water-soluble flavonoid from the catechin group, and is found in abundance in green tea (Figure 1A). EGCG possesses a wide spectrum of biological activities, including anti- and pro-oxidant, anti-inflammatory, anticancer and anti-infective (antiviral, antibacterial and antifungal) activities [1,2,3,4]. These activities of EGCG have been observed at doses that are safe for humans [5,6,7,8], shown to stimulate numerous clinically oriented studies of EGCG.

The mechanisms of EGCG action in mammalian cells include interactions with cell surface receptors, penetration into cytoplasm and interaction there with different signaling pathways, penetration into the nucleus and the modulation of gene expression [9]. The modulation of gene expression with EGCG is not accompanied by genotoxicity [10], and is assumed to be associated with the inhibition of different transcription factors, DNA methyltransferase and class I histone deacetylases [9,11,12]. Nucleus-associated mechanisms of EGCG action include the poisoning of topoisomerases I and II and the inhibition of poly(ADPribose)polymerase 1 (PARP1) [9,13,14,15,16]. PARP1 is a sensor of DNA damage, and recruits DNA repair proteins to DNA lesions [17]. It is considered to be a target for the treatment of malignant neoplasms with homologous recombination deficiency [18]. PARP1 is involved in the regulation of gene expression, increasing the activity of transcription factors and acting on the proteins participating in methylation of DNA [19].

It was proposed that EGCG penetrates into the nucleus and binds to DNA and RNA [20]. In vitro experiments confirmed that EGCG interacts with dsDNA, ssDNA and RNA [20]. The binding constant of the dsDNA–EGCG complex was measured to be (2.3 ± 0.8) × 10^5^ M^−1^ [21]. According to molecular dynamics simulations, the EGCG interaction with dsDNA occurs due to intercalation through the major groove, and is characterized by a low DNA sequence specificity [22]. The intercalation of EGCG into dsDNA was confirmed experimentally [23,24]. At the same time, the binding of EGCG to nuclear DNA in living cells was not directly demonstrated, and the interaction of EGCG with chromatin was not systematically investigated.

To fill this gap, the present work focuses on the study of the chromatin-targeted activity of EGCG in vitro, namely, on the EGCG interaction with nucleosomes, chromatosomes and nucleosome complexes with PARP1. It is found that EGCG considerably reorganizes these structural units of chromatin and modulates the structure of PARP1–nucleosome complexes in vitro. Similar interactions could occur in vivo, since it is shown here that EGCG effectively penetrates into the nuclei of living cells and binds to DNA. Our data suggest that the reorganization of chromatin structures with EGCG could affect DNA repair, replication and transcription processes, and contribute to the regulation of gene expression.

## 2. Results

### 2.1. Binding of EGCG to DNA in Living Cells

Earlier, it was reported that Hoechst 33258, which binds to the minor groove of DNA, can be displaced from complexes with DNA in solution using different polyphenols, which are supposed to be either intercalators or minor groove-binding agents [25]. Hoechst 33342 is similar to Hoechst 33258 in structure and in the mode of binding to DNA [26], but Hoechst 33342 penetrates easier in living cells, and its fluorescence has been predominantly observed in the nuclei in complexes with DNA (Figure 1C,D) [27]. Hoechst 33342 fluoresces in the blue region, while EGCG fluoresces in the UV range, and the excitation of Hoechst 33342 fluorescence at the 405 nm wavelength does not result in EGCG excitation. Taking into account these properties of Hoechst 33342 and EGCG, the fluorescence microscopy approach, which is based on the competition between Hoechst 33342 and EGCG for binding to DNA in living cells, was applied to evaluate the ability of EGCG to penetrate within the nuclei of cells and to interact with the DNA there.

Our study showed that exposure of HEK293 cells, which were preincubated with Hoechst 33342, to EGCG led to a concentration-dependent decrease in the fluorescence of DNA–Hoechst 33342 complexes (Figure 1B–F), suggesting that EGCG penetrated the nuclei of the living cells, interacted with DNA and displaced Hoechst 33342.

The results of our study were consistent with previously published data on the intranuclear penetration of EGCG [28] and provided direct evidence of the interaction of EGCG with DNA in living cells.

### 2.2. Experimental Approaches for Analysis of EGCG–Nucleosome Interaction

Since DNA in the nucleus is organized in nucleosomes, the question arises about the effect of EGCG interaction with DNA on the structure of nucleosomes and the complexes of nucleosomes with nuclear proteins. To address this question, fluorescently labeled mononucleosomes were assembled and studied using the electrophoretic mobility shift assay (EMSA) and Förster resonance energy transfer (FRET) fluorescent microscopy of single particles (spFRET microscopy).

**Figure 2:**
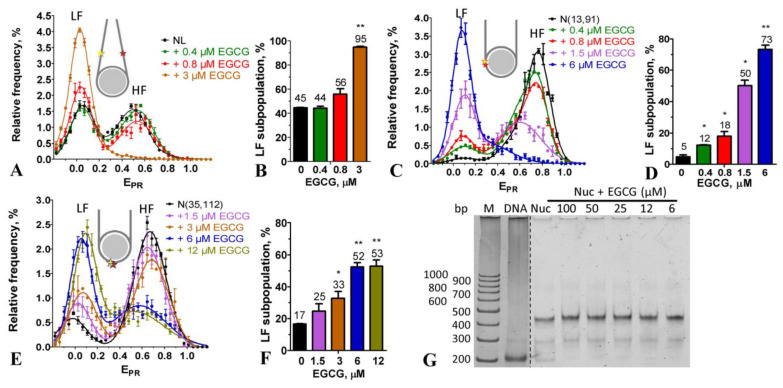
EGCG interaction with nucleosomes. (**A**,**C**,**E**) Frequency distributions of nucleosomes NL (**A**), N(13, 91) (**C**) and N(35, 112) (**E**) with E_PR_ value in the absence and presence of different concentrations of EGCG (mean ± SEM, *n* = 3). Insets—schematic structures of the studied nucleosomes with the positions of Cy3 and Cy5 labels marked with yellow and red asterisks, respectively. (**B**,**D**,**F**) Content of the low FRET (LF) subpopulation of nucleosomes NL (**B**), N(13, 91) (**D**) and N(35, 112) (**F**) at different EGCG concentrations. The LF subpopulations were calculated on the basis of the corresponding E_PR_ profiles. *—*p* < 0.05; **—*p* < 0.005 as compared to nucleosomes without EGCG. (**G**) Analysis of the EGCG–nucleosome complexes with nondenaturing PAGE. M—marker; Nuc—nucleosomes.

Mononucleosomes were assembled using octamers of human histones and DNA containing the 147 bp nucleosome positioning sequence 603 (NPS603), which was flanked with two 20 or 40 bp DNA linkers (see insets in Figure 2A,C,E). Three types of fluorescently labeled nucleosomes were prepared. Nucleosomes N(13, 91) and N(35, 112) with 20 bp linkers had fluorescent labels (a donor–acceptor pair of labels, Cy3 and Cy5) within the nucleosome core at positions 13 and 91 bp (N(13, 91)) or 35 and 112 bp (N(35, 112)) from the beginning of the NPS603. Cy3 and Cy5 labels were located close to each other on the neighboring gyres of nucleosomal DNA and supported an efficient FRET [29]. Nucleosomes NL with two 40 bp linkers had fluorescent labels in linkers at positions 10 bp before and 10 bp after the NPS603. The different positions of the pair of labels enabled the analysis of structural changes in the corresponding regions of a nucleosome with the FRET technique. The application of the spFRET microscopy provided the detection of structurally different subpopulations of nucleosome complexes with EGCG and PARP1. The technique was based on the measurement of FRET efficiency from single nucleosomes diffusing through the focus of laser beam in the highly diluted solution.

FRET was sensitive to structural changes when the distance between the Cy3 and Cy5 labels varied from 4 to 9 nm. To further characterize large-scale structural changes in the nucleosomes and their complexes with EGCG and PARP1, the EMSA was used.

### 2.3. Effect of EGCG on the Structure of Nucleosomes

NL nucleosomes were used to study the effect of EGCG on the conformation of nucleosomes within the linker DNA region. In agreement with previously published data [30], the spFRET microscopy revealed two subpopulations of NL, which differed in the conformation of the linker DNA. These subpopulations of nucleosomes were recognized in the E_PR_ profile (the graph showing the distribution of nucleosomes with the FRET efficiency) as low (LF) and high FRET (HF) peaks and were characterized by the different distances between DNA helices in the linker region, where the fluorescent labels were located. The binding of EGCG to NL noticeably affected the conformation of linker DNA at the EGCG concentration higher than 0.8 µM (Figure 2A) and led to a decrease in the HF subpopulation of NL with a concomitant increase in the LF subpopulation (Figure 2A,B). Thus, the binding of EGCG to the nucleosome increased the distance between the DNA helices in the linker region.

The study of the N(13, 91) and N(35, 112) nucleosomes showed that the effect of EGCG on the nucleosome structure was not limited to the linker region and extended to the core region (Figure 2C,E). For both the N(13, 91) and N(35, 112) nucleosomes, the E_PR_ profiles were characterized by a dominant HF peak and a small LF peak in the absence of EGCG. The nucleosomes belonging to the HF peak had an intact structure with a close arrangement of DNA gyres on the histone octamer in the area of the fluorescent labels. LF subpopulations of the N(13, 91) and N(35, 112) nucleosomes could combine subnucleosomes, free nucleosomal DNA and nucleosomes with partially uncoiled nucleosomal DNA. “Breathing” or the temporal spontaneous unwinding of 10–20 bp of nucleosomal DNA from the histone octamer near the nucleosome boundary [31] could also be detected in the case of N(13, 91), with the labels positioned close to the nucleosome boundary. Note that the proportion of the LF nucleosomes could vary from batch to batch of the assembled and purified nucleosomes.

The addition of EGCG to the nucleosomes resulted in an increase in the LF subpopulation of N(13, 91) and a decrease in their HF subpopulation that was accompanied by a shift in the HF peak maximum in the E_PR_ profile (Figure 2C). The alterations of the E_PR_ profile of N(13, 91) appeared at 0.4 µM and were more pronounced at higher EGCG concentrations (Figure 2C,D). The shift in the HF peak to lower the E_PR_ values reflected a change in the conformation of nucleosomal DNA near the nucleosome boundary, which was accompanied by an increase in the distance between DNA gyres. This change was especially pronounced at ca. 1.5 µM EGCG (Figure 2C). An increase in the LF subpopulation could correspond to either the uncoiling of nucleosomal DNA from the histone octamer that involved at least the 40 bp region at the beginning of the NPS603 (as estimated previously [32]) and/or the disassembly (partial or total) of the nucleosomes. An increase in EGCG concentration to 6 µM resulted in the conversion of the majority of the N(13, 91) nucleosomes into an LF subpopulation (Figure 2C,D).

Similar structural changes were observed in the N(35, 112) nucleosomes interacting with EGCG (Figure 2E,F); however, they occurred at higher concentrations of EGCG as compared to the N(13, 91) nucleosomes (Figure 2C,D), suggesting that a higher EGCG concentration was required to disturb the nucleosome structure in the region located 35 bp away from the beginning of the NPS603. An increase in EGCG concentration higher than 6 µM did not lead to additional changes in the structure of nucleosomes in the 35 and 112 bp regions (Figure 2E,F and Figure S1), indicating that the saturation of EGCG binding to the nucleosomes occurred at these concentrations. The data were, in general, in agreement with the previously reported value of the dissociation constant of the EGCG–DNA complex (4.3 µM) [21].

According to the spFRET data, ca. 52% of the nucleosomes had a disturbed conformation of nucleosomal DNA with an increased distance between DNA gyres in the region localized 35 bp away from the beginning of the NPS603 at the EGCG concentration higher by 6 µM. In the remaining nucleosomes, a large-scale uncoiling of the nucleosomal DNA from the histone octamer occurred, involving at least 60 bp of the NPS603 from either end of the nucleosome (as estimated previously [32]).

**Figure 3:**
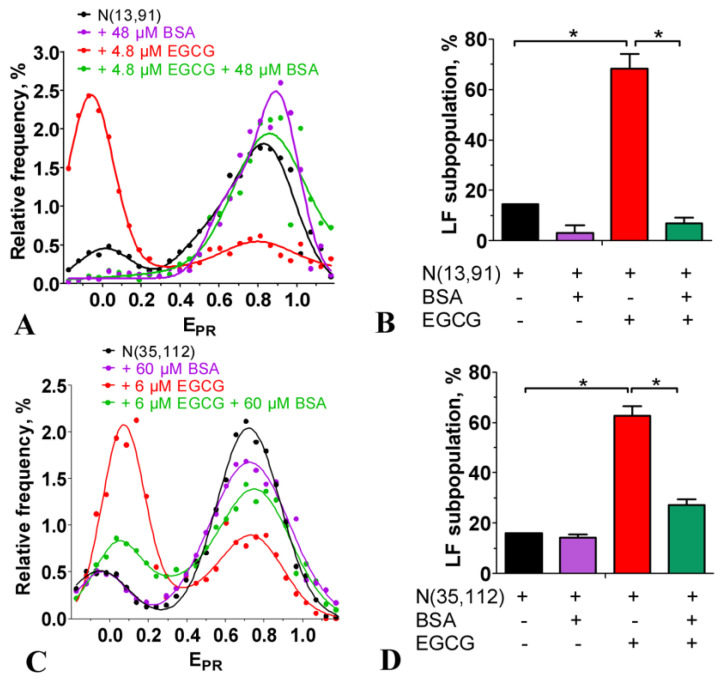
Reversibility of the EGCG-induced changes in a nucleosome structure. (**A**,**C**) Typical frequency distributions of nucleosomes N(13, 91) (**A**) and N(35, 112) (**C**) by E_PR_ value in the absence and presence of EGCG (4.8 or 6 µM) and BSA (48 or 60 µM). (**B**,**D**) Content of the low FRET (LF) subpopulation of nucleosomes N(13, 91) (**B**) and N(35, 112) (**D**) in the absence and presence of EGCG and BSA. The LF subpopulations were calculated from the corresponding E_PR_ profiles. *—*p* < 0.05.

The EMSA showed that the incubation of nucleosomes with up to 100 µM of EGCG was not accompanied by the dissociation of DNA from the histone octamer or by the formation of subnucleosomes that had lost some histones (Figure 2G). This conclusion was confirmed with the spFRET analysis, showing that after adding an excess of BSA, which bound EGCG [33], the EGCG–nucleosome complexes dissociated, and the released nucleosomes restored the intact structure (Figure 3). BSA itself did not affect the nucleosome structure (Figure 3). Taken together, the data suggest that the structural changes induced through the use of EGCG in the nucleosomes occurred without the loss of histones and were reversible.

### 2.4. Effect of EGCG on the Structure of Chromatosomes

**Figure 4:**
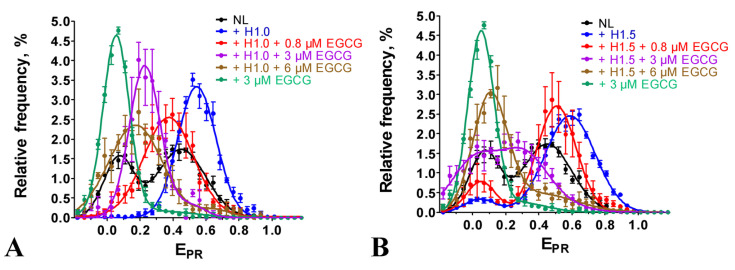
EGCG interaction with chromatosomes formed in the presence of linker histones H1.0 (**A**) and H1.5 (**B**). Frequency distributions by E_PR_ value (mean ± SEM, *n* = 3) are shown for nucleosomes NL and their complexes with EGCG (3 μM), as well as for chromatosomes and their complexes with EGCG (0.8, 3 and 6 μM).

The linker histone H1 bound to the nucleosomes and brought together the DNA linkers, forming chromatosomes [34]. In agreement with the data reported earlier [30], this structural transition was detected in the E_PR_ profiles of chromatosomes formed with NL as an appearance of the single HF peak, having a maximum at 0.55 or 0.59, depending on the type of the linker histone, H1.0 or H1.5, respectively, instead of two peaks (maxima at 0.07 and 0.45), characteristic for intact nucleosomes (Figure 4). The binding of EGCG to the chromatosomes induced a disturbance in the DNA conformation in the linker region observed as a shift in the peak in the E_PR_ profile to the lower E_PR_ values (Figure 4). This shift was more pronounced at higher EGCG concentrations. Thus, at 6 µM EGCG (saturation of EGCG binding to nucleosomes), the peak maximum was localized at 0.17 or 0.11 for H1.0- or H1.5-containing chromatosomes, respectively (Figure 4). Since the maximum of the E_PR_ profile of the EGCG–NL complexes was at the lower E_PR_ value (0.07, Figure 4) and the differences in the positions of the peak maxima of the EGCG–NL and EGCG–chromatosome complexes were significant (*p* < 0.05), the disturbance in the chromatosome structure due to EGCG, leading to an increase in the distance between the DNA helices in the linker region, was unlikely to be accompanied by the dissociation of the linker histones H1.0 and H1.5, even at the highest concentration of EGCG.

### 2.5. Effect of EGCG on the PARP1–Nucleosome Complexes

Since EGCG is an inhibitor of PARP1 [16], we studied the effect of EGCG on the interaction of PARP1 with nucleosomes.

**Figure 5:**
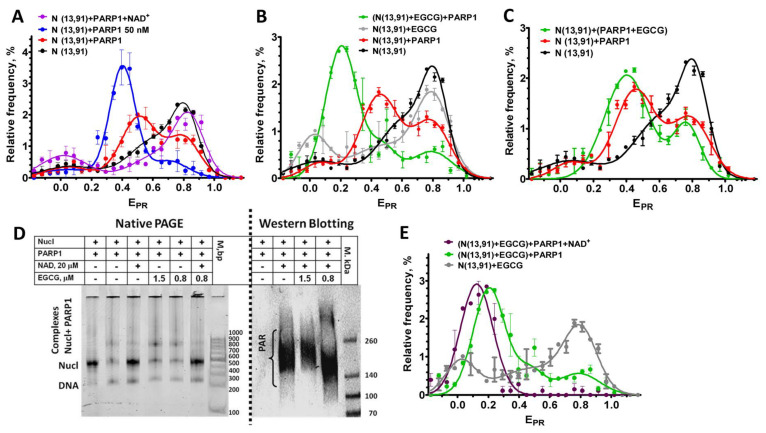
Effect of EGCG on the interactions of nucleosomes with PARP1. (**A**) E_PR_ profiles of nucleosomes N(13, 91) and their complexes with PARP1 (20 or 50 nM) or with PARP1 (20 nM) after addition of NAD^+^ (20 µM). (**B**,**C**) E_PR_ profiles of nucleosomes N(13, 91) and their complexes, either with EGCG (0.8 µM) or with PARP1 (20 nM), or with PARP1 (20 nM) in the presence of EGCG (0.8 µM). PARP1 was added to nucleosomes preincubated (15 min) with EGCG (**B**) or simultaneously with EGCG (**C**). (**D**) Analysis of the complexes of nucleosomes with PARP1, EGCG and NAD^+^ with nondenaturing PAGE or with Western blotting with the use of antibodies to PAR. (**E**) E_PR_ profiles of the complexes of nucleosomes N(13, 91) with either EGCG (0.8 µM) or with EGCG (0.8 µM) and PARP1 (20 nM) or with PARP1 and EGCG after addition of NAD^+^ (20 µM). (**A**–**C**,**E**) Data are mean ± SEM (*n* = 3).

In agreement with the published data [35], PARP1 binding to the nucleosomes resulted in an overall increase in the distance between DNA gyres on the histone octamer that was observed as a shift in the HF peak, a characteristic of the intact conformation of nucleosomal DNA, to the middle FRET region (MF, E_PR_~0.45) in the E_PR_ profile (Figure 5A). Binding to the nucleosome activated the enzymatic activity of PARP1, and the addition of the substrate, NAD^+^, resulted in the polyADPrybosylation (PARylation) of histones and PARP1 itself [35]. Eventually, the PARylated PARP1 dissociated from the nucleosome because of the accumulation of a negative charge, and the released nucleosomes assumed the native conformation of nucleosomal DNA, characterized by the reappearance of the characteristic HF peak in the E_PR_ profile (Figure 5A) [35].

To study the effect of EGCG on the PARP1–nucleosome interaction, including changes in the PARP1 affinity for the nucleosome, the concentration of PARP1 (20 nM) was selected at which ca. 65% of nucleosomes was recruited in the complex (Figure 5A–C). A concentration of EGCG (0.8 µM) was selected, which was eightfold less than the concentration of the saturated binding (ca. 6 µM), but it still induced noticeable changes in the E_PR_ profile of the N(13, 91) nucleosomes (Figure 5B). The addition of PARP1 (20 nM) to the preformed EGCG–nucleosome complexes resulted in the appearance of a new peak (E_PR_ = 0.2) in the E_PR_ profile that was positioned between the LF peak of the EGCG–nucleosomes complexes and the MF peak of the PARP1–nucleosome complexes (Figure 4B). This peak of the EGCG–nucleosome–PARP1 complexes comprised ca. 67% of the detected particles, indicating that the fraction of nucleosomes that formed the complexes with PARP1 did not change in the presence of EGCG. Thus, EGCG did not disturb the binding of the nucleosomes to PARP1, but strongly affected the structure of the complexes, further increasing the distance between DNA gyres on the histone octamer as compared to the PARP1–nucleosome complex. An increase in the EGCG concentration up to 10 µM also did not reduce the binding of PARP1 to the nucleosomes (see Figure 5D and Figure S2).

When PARP1 (20 nM) was preincubated with EGCG (0.8 µM) and added to the nucleosomes, the effect of EGCG on the structure of the complexes was less pronounced, with a smaller increase in the distance between the DNA gyres as compared to the preformed EGCG–nucleosome complexes interacting with PARP1 (compare Figure 4B,C). The data suggest that PARP1 bound to the nucleosomes faster than EGCG, and, being bound to nucleosomes, interfered with the binding of EGCG to nucleosomal DNA.

The addition of NAD^+^ to the EGCG–nucleosome–PARP1 complexes resulted in the dissociation of PARP1 from the nucleosomes (see native PAGE in Figure 5D). This dissociation was induced by the autoPARylation of PARP1 that occurred efficiently at sub- and low-micromolar concentrations of EGCG (see WB in Figure 5D). An increase in the EGCG concentration to 10 µM did not interfere with the NAD^+^-induced dissociation of PARP1 from the nucleosomes, indirectly indicating the preservation of the autoPARylation activity of PARP1, even at the saturating concentration of EGCG (Figure S2). Therefore, the EGCG interaction with the nucleosomes did not prevent the PARP1 from binding to nucleosomal DNA, which was required for the enzyme activation, and EGCG did not inhibit the catalytic activity of PARP1.

The dissociation of the PARylated PARP1 from the nucleosomes in the presence of EGCG did not restore the E_PR_ profile that was observed before the PARP1 binding to EGCG–nucleosome complexes (Figure 5E). Instead, a single peak was observed with the maximum having shifted to the E_PR_ value of 0.13, indicating that all the nucleosomes adopted a similar conformation with the increased distance between the DNA gyres. It could be supposed that the association of EGCG with the additional binding sites that became available in the nucleosomes reorganized through PARP1 enhanced the effect of EGCG on the structure of the released nucleosomes.

## 3. Discussion

The limited bioavailability and reduced absorption rate of EGCG resulted in a limited (micromolar) concentration of EGCG in the plasma and tissues [36]. Therefore, the achievable concentrations of EGCG in the cells could determine the spectrum of the biological effects of EGCG and its effectiveness.

Our experiments showed that EGCG penetrated into the nuclei of the cells and bound there with DNA (Figure 1). The nuclear concentration of EGCG was unknown, but could be roughly estimated from our experiments. The intranuclear concentration of EGCG was high enough to compete with Hoechst 33342 for binding to DNA even at the low-micromolar extracellular concentrations of EGCG (Figure 1B). The bright fluorescent signal of Hoechst 33342 in cells indicated that the concentration of the dye bound to DNA was higher than 0.1 µM. The binding constants of DNA to dyes of the Hoechst family and EGCG were ca. 2 × 10^8^ M^−1^ [37] and 2.3 × 10^5^ M^−1^ [21]. Therefore, to displace Hoechst 33342 from the complexes with DNA, the intranuclear concentration of EGCG would need to be a thousand times higher than that of Hoechst 33342 and achieve ca. 100 µM. This high intranuclear accumulation of EGCG explained the previous observation that “nucleic acids extracted from EGCG-treated human cancer cells were catechin colored” [20]. The very high distribution coefficient (the ratio of the intracellular to extracellular concentration) of EGCG is probably the reason why the consumption of green tea is useful in the case of various human diseases [38,39,40] in spite of the limited bioavailability of EGCG. EGCG effectively interacted with nucleosomal DNA at sub- and low-micromolar concentrations (Figure 2 and Figure 4), while the inhibition of DNA methyltransferase occurred at sub-micromolar concentrations of EGCG [9], and class I histone deacetylases were inactivated through the use of EGCG at 50–200 µM [12]. The poisoning of topoisomerases I and II required the interaction of EGCG with DNA and was realized at micromolar concentrations of EGCG [9,13,14,15]. The intercalation in DNA created an increased local concentration of EGCG, which may have enhanced the inhibition of enzymes interacting with chromatin. Our experiments with BSA (Figure 3) showed that EGCG intercalated into DNA could easily migrate from DNA to EGCG-binding proteins.

Our results showed that EGCG could affect the activity and interaction of certain enzymes, for example, PARP1 to nucleosomes in a complex way. Thus, although the affinity of PARP1 to nucleosomes did not reduce in the presence of EGCG, the catechin caused the modulation of the structure of the PARP1–nucleosome complex (Figure 5), which, in turn, could affect the assembly of the DNA repair complex, the process triggered through the PARP1-mediated synthesis of PAR polymers.

The effect of EGCG on the catalytic activity of PARP1 was intricate. PARP1 activity did not change at sub- and low-micromolar concentrations of EGCG, as probed with WB and EMSA, which mainly addressed the autoPARylation activity of PARP1 (Figure 5). Previously, the inhibition of PARP1 with EGCG was observed in vitro using the assay based on the analysis of the PARylation of immobilized histones, i.e., the trans-PARylation activity of PARP1 [16]. This discrepancy probably occurred because EGCG differentially affected the trans- and auto-PARylation activities of PARP1, similar to some other factors reported earlier [41].

Nucleosomes and chromatosomes are basic structural units of chromatin, and our data showed that EGCG reorganized them in vitro at sub- and low-micromolar concentrations (Figure 2 and Figure 4). EGCG-induced conformational changes occurred in both linker and nucleosomal DNA and led to a large-scale uncoiling of nucleosomal DNA from the histone octamer, which, depending on the EGCG concentration, involved up to 60 bp from each of the two nucleosome boundaries. This reorganization of nucleosomes was not accompanied by the dissociation of either DNA or core histones, and was completely reversible after EGCG dissociation from the nucleosome (Figure 2G and Figure 3). This suggested a low probability of irreversible structural changes caused through the use of EGCG in chromatin and, at least in part, might explain the lack of EGCG genotoxicity [10]. The reorganization of chromatosomes using EGCG, which very likely occurred without the dissociation of the linker histone (Figure 4), suggested that the tight packing of heterochromatin may also have been disrupted due to EGCG in the cells.

Since no more than one EGCG molecule bound per 3–6 base pairs of DNA [20], the maximum number of EGCG molecules bound to a nucleosome assembled on DNA of 227 bp length was 38–75, and it was achieved at the saturation of binding at 6 µM EGCG. Assuming a linear relationship between the concentration and the number of bound EGCG molecules, the noticeable disturbance in the nucleosome and chromatosome structure, which was observed at ca. 1 µM EGCG (Figure 2 and Figure 4), occurred at the number of bound EGCG molecules of 6–12 per nucleosomes (1 EGCG molecule per 18–36 bp). Can the number of DNA-bound EGCG molecules be high enough to destabilize the structure of chromatin in cells? The average intranuclear concentration of DNA is ca. 10 mg/mL or 15 mM (in base pairs), assuming that all DNA is double-stranded [42]. On the basis of the binding constant of the dsDNA–EGCG complex, one could calculate that the equilibrium totally shifted to the DNA-bound state even at nanomolar intranuclear concentrations of EGCG, and even if no more than 0.1% of nuclear DNA was accessible for the interaction with the catechin. Therefore, the destabilization of the chromatin structure would occur at the nuclear concentration of EGCG of 400–800 µM or 4–8 µM, depending on whether 100 or 1% of DNA in the chromatin was accessible for the interaction, respectively. Since the nuclear concentration of EGCG could be as high as 100 µM (see above), the EGCG-induced destabilization of the chromatin structure was a very likely mechanism of EGCG action. The EGCG-induced reorganization of nucleosomes and chromatosomes could facilitate access to protein factors involved in DNA repair, replication and transcription to nucleosomal DNA and, thus, contribute to the observed modulation of gene expression [9].

## 4. Materials and Methods

### 4.1. Cellular Experiments

HEK293 cells (the Russian collection of cell cultures, the Institute of Cytology of the Russian Academy of Sciences, Saint Petersburg, Russia) were grown in Dulbecco’s modified Eagle’s medium DMEM/F12 (Paneco, Moscow, Russia) supplemented with 10% fetal bovine serum (HyClone, Logan, UT, USA) and 2 mM L-glutamine (complete medium) and transplanted two times a week. A day before the experiments, the cells were transferred into 96-well plates ((2.0 ± 0.4) × 10^4^ cells per well) and grown in the complete medium (37 °C, 5% CO_2_). The cells were incubated with 1 µM Hoechst 33342 for 3.5 h and further incubated with EGCG (2.5–25 µM) for 2.5 h in the complete medium. The control cells were incubated with 1 µM Hoechst 33342 for 6 h in the complete medium. The cells were imaged with ZOE Fluorescent Cell Imager (Bio-Rad, Singapore). Fluorescence was excited and recorded in the 335–375 and 415–451 nm spectral ranges, respectively. Fluorescent images of the cells were analyzed with Image J software (ver. 1.54f29, National Institute of Health, Bethesda, MD, USA) using the “Analyze particles” plugin. The sampling size varied from 400 to 600 cells. The experiments were repeated in triplicate.

### 4.2. DNA

The DNA templates were obtained through a polymerase chain reaction (PCR) using a plasmid containing the 603–42A nucleosome positioning sequence [43] and fluorescently labeled primers. The DNA templates 187 bp in length were obtained using primers published previously [44].

The 227 bp DNA template was obtained in a two-step PCR, as described earlier [30], using the forward 5′-CACCGGCACGAGGGCCCGGTTC-3′ primer and the reverse 5′-ACTTTCTGGCAAGAAAATGAGCT-3′ primer in the first step and the following fluorescently labeled primers in the second step:

foward-5′-ACACGGCGCACTGCCAACCCAAACGACACCT[Cy3-dT]GCACGAG-3′;

reverse-5′-TAAGGCGAATTCACAACTTTTTGGCAAGAAT[Cy5-dT]ATGAGCT-3′. 

The purification and extraction of the PCR products from 2% agarose gel were performed using QIAquick Gel Extraction Kit (Qiagen, Venlo, The Netherlands).

### 4.3. Proteins

Recombinant human histones were obtained in a bacterial expression system. The canonical genes of human histones H2A, H2B, H3 and H4 were cloned into a pET-3-based vector and expressed in *E. coli* cells at 37 °C. The expression of histone H4 was carried out in the strain Rosetta 2(DE3). Histones H2A, H2B and H3 were expressed in the strain BL21(DE3). An overnight culture was inoculated into Luria broth (LB) medium with 100 µg/mL ampicillin (and 24 µg/mL chloramphenicol for Rosetta 2(DE3)). Isopropyl-beta-D-thiogalactopyranoside (IPTG, 0.4 mM) was added to the cells when their optical density reached 0.6 optical units/cm at 600 nm. Two hours after the induction, the cells were harvested through centrifugation (3200× *g*, 30 min), washed with water and frozen. The purification of individual histones was performed using the procedure described earlier [45] with the option of the sequential stacking of HiTrap Q FF (5 mL) and HiTrap SP HP (5 mL) columns.

The expression vector based on the pET3 plasmid containing the gene of human linker histone H1.5 was kindly provided by S. Dimitrov. Rosetta2(DE3) *E. coli* cells were transformed with this vector through electroporation. An overnight culture of transformed cells was inoculated into liquid LB medium containing 100 µg/mL ampicillin. The cells were grown at 37 °C until an optical density of 0.6 optical units/cm at 600 nm. Protein expression was induced by adding IPTG (0.5 mM), and after 4 h of incubation, the cells were harvested through centrifugation (3900× *g* for 20 min at 4 °C), washed with PBS buffer and shock-frozen with liquid nitrogen. The purification of H1.5 was performed using the procedure described earlier [45] using sequentially stacked HiTrap Q FF (5 mL) and HiTrap SP FF (5 mL) ion exchange columns. Protein-containing fractions were collected, concentrated and dialyzed against a SAU-1000 buffer (400 mM NaOAc, 10 mM EDTA, 100 mM lysine-HCl, 1 M NaCl, 6 M urea, 5 mM β-mercaptoethanol (β-ME)). The protein solution was applied onto a Hi-Prep Sephacryl 16/60 S200 HR gel filtration column equilibrated with the SAU-1000 buffer. The protein elution was performed with the same buffer. Protein-containing fractions were collected, dialyzed against 20 mM Tris-HCl pH 8.0 at 4 °C for 24 h and concentrated.

Recombinant linker histone H1.0 from *Xenopus laevis* was expressed in *E. coli* as described earlier [46].

The expression plasmid pET-28-PARP1-bearing gene of human PARP1 fused with hexahistidine tag at the N-terminus was kindly provided by J.M. Pascal. The plasmid was transformed into *E. coli* cells (strain Rosetta2 (DE3) pLysS). The cells were grown on LB agar plates containing 50 µg/mL kanamycin and 35 µg/mL chloramphenicol overnight at 37 °C. Then, a single colony was inoculated into LB media supplemented with the same antibiotics, grown overnight at 37 °C and transferred into a large volume of LB medium (1–4.5 L) at a hundredfold dilution. The cells were cultivated at 37 °C in the presence of kanamycin (50 µg/mL), chloramphenicol (35 µg/mL) and benzamide (10 mM) until an optical density of 0.8–1.0 optical units/cm at 600 nm. After chilling the cells on ice, PARP1 synthesis was induced with 0.2 mM IPTG in the presence of 0.1 mM ZnCl_2_. The cells were incubated at 16 °C for 16 h, centrifuged at 3900× *g* for 30 min, resuspended in a storage buffer (25 mM HEPES pH 8.0, 500 mM NaCl, 0.5 mM tris(2-carboxyethyl)phosphine (TCEP), 10 mM benzamide) and frozen at −80 °C or used for protein purification.

All the further purification steps were accomplished at 4 °C on ice. The cells were lysed using an ultrasonic disintegrator with the preliminary addition of 0.1% NP-40 and protease inhibitors (1 mM PMSF, 0.5 µg/mL leupeptin, 0.7 µg/mL pepstatin A, 0.5 µg/mL antipain, protease inhibitor cocktail P2714 (Sigma-Aldrich, Merck, Germany) and 1 mM benzamidine). The lysate was centrifuged at 18,000× *g* for 1.5 h, filtered (0.22 µm) and applied to the HiTrap chelating column (Cytiva, Marlborough, MA, USA) for affinity chromatography. All the chromatography steps were performed with the AKTA Purifier chromatography system (Cytiva, Marlborough, MA, USA). Next, the column was washed sequentially with three buffers (25 mM HEPES pH 8.0, 0.5 mM TCEP, 20 mM imidazole, protease inhibitors) containing 500, 1000 and, again, 500 mM NaCl. PARP1 was eluted with a buffer containing 25 mM HEPES pH 8.0, 500 mM NaCl, 0.5 mM TCEP and 250 mM imidazole. The eluate was diluted with a salt-free buffer (50 mM Tris-HCl pH 7.0, 1 mM EDTA, 0.1 mM TCEP) 2.5 times and filtered. Then, the sample was applied to the HiTrap heparin column (Cytiva, USA) and washed with buffer A (50 mM Tris-HCl pH 7.0, 200 mM NaCl, 1 mM EDTA, 0.1 mM TCEP) and an increasing gradient (from 0 to 100%) of buffer B (50 mM Tris-HCl pH 7.0, 1 M NaCl, 1 mM EDTA, 0.1 mM TCEP). Fractions containing PARP1 (analyzed with SDS-PAGE) were pooled, concentrated with Amicon Ultra 4 unit and centrifuged to remove insoluble components. Finally, the protein was applied to a HiPrep 16/60 Sephacryl S-200 HR gel filtration column and eluted with a gel filtration buffer (25 mM HEPES pH 8.0, 150 mM NaCl, 1 mM EDTA, 0.1 mM TCEP). The purity and homogeneity of the obtained fractions were analyzed with SDS-PAGE. PARP1-containing fractions were concentrated, flash-frozen and stored at −80 °C.

### 4.4. Nucleosomes

The assembly of histone octamers was performed according to the protocol of denaturation-refolding followed by gel filtration on Superdex 200 [47]. The purity of the histone octamer was analyzed using sodium dodecyl sulfate–polyacrylamide gel electrophoresis. The concentration of the histone octamer was measured with UV spectrophotometry.

Histone octamers and DNA templates were used to assemble nucleosomes in the course of dialysis against the decreasing concentration of NaCl, as described earlier [48]. The assembled nucleosomes were analyzed with electrophoresis in nondenaturing 4.5% polyacrylamide gel (acrylamide:bisacrylamide 39:1; 0.5× TBE buffer, pH 8.0).

For experiments with PARP1, the nucleosomes were isolated from the gel through extraction in a buffer containing 10 mM HEPES-NaOH (pH 8.0), 0.2 mM EDTA, 0.2 mg/mL bovine serum albumin (BSA) and stored at 4 °C.

### 4.5. Preparation of Complexes for spFRET Analysis

EGCG (Sigma-Aldrich, Merck, Rahway, NJ, USA) was dissolved in 100% DMSO at the concentration of 5 mM and stored at −20 °C. The preparation of samples for the spFRET analysis was performed according to the protocols published previously [35,44]. Briefly, EGCG (0.4–50 µM) was mixed with nucleosomes (1 nM) in a buffer (150 mM KCl, 5 mM MgCl_2_, 1 mM β-ME, 20 mM Tris-HCl, pH = 7.5) and incubated for 20 min at room temperature (RT). In the experiment with BSA, BSA (48 or 60 µM) was added to the preformed EGCG–nucleosome complexes and the mixture was incubated for 20 min at RT. In the experiments with linker histones, the nucleosomes were incubated with H1.0 (15 nM) or H1.5 (7.4 nM) for 15 min in low-adhesion tubes; half of the solution was mixed with EGCG (0.8–6 μM) and was further incubated for 20–30 min at RT. The DMSO concentration in the samples with nucleosomes was less than 1% and did not affect the structure of the nucleosomes, as verified with the spFRET microscopy (Figure S3).

In experiments with PARP1, EGCG (0.8 µM) was mixed with nucleosomes (1 nM) in the TB150 buffer (20 mM Tris-HCl (pH 7.9), 5 mM MgCl_2_, 150 mM KCl, 1 mM β-ME) and incubated for 15 min at RT; PARP1 (20 or 50 nM) was added to this mixture or to the nucleosomes (1 nM) and the samples were incubated for 30 min at RT. The preincubated samples containing PARP1 were mixed with NAD+ (20 µM) and incubated for 45 min at RT. Alternatively, PARP1 (20 nM) was mixed with EGCG (0.8 µM), incubated for 15 min at RT and then incubated with nucleosomes (1 nM) for 30 min at RT.

### 4.6. spFRET Microscopy

The spFRET measurements were carried out using the LSM 710-Confocor3 system (Carl Zeiss, Aalen, Germany) with the C-Apochromat water-immersion lens (40×, NA1.2). The samples were placed into the 12-well silicon chamber (ibidi Gmbh, Martinsried, Germany) attached to a cover glass. Details of the measurements were described earlier [12,48]. The fluorescence of the single nucleosomes and their complexes diffusing through the laser focus was excited at the 514.5 nm wavelength (0.2 μW) and recorded in the 530–635 (Cy3) and 635–800 nm (Cy5) ranges during 10 min at the 3 ms integration time. The confocal diaphragm was set to 1 Airy disk. The proximity ratio (E_PR_) was calculated for each detected particle as described earlier [48]. The data samples were presented as frequency distributions of nucleosomes in the E_PR_ value.

The E_PR_ profiles were fitted as a superposition of one, two or three Gaussian peaks. The selection of the number of Gaussian peaks was determined after a comparison of alternative variants through the root-mean-square deviation. The content of the specific subpopulations of nucleosomes was calculated as the ratio of the areas under specific Gaussian peaks to the total area under the E_PR_ profile (as a percentage). Results of the analysis were averaged.

### 4.7. Electrophoresis

The preparation of samples for electrophoresis was performed using the protocols published earlier [35,44]. Briefly, for the electrophoresis aimed to study the integrity of the EGCG–nucleosome complexes, EGCG (6–100 µM) was added to nucleosomes (60 nM) in 0.01 M buffer for dialysis (10 mM NaCl, 10 mM Tris-HCl (pH 7.9), 0.1% NP40, 0.2 mM EDTA, 5 mM β-ME), incubated for 20 min, mixed with sucrose (final concentration 10%) and loaded into the 4% polyacrylamide gel. Electrophoresis was performed in the HE buffer (10 mM HEPES-NaOH pH 8.0, 0.2 mM EDTA) at +4 °C at 80 V.

In the PARP1-related studies, nucleosomes (2–3 nM) were preincubated with EGCG (0.8–10 µM, 15 min, 25 °C) and further incubated with PARP1 (20 or 50 nM) or with PARP1 and NAD+ (20 μM) in the TB150 buffer (45 min, 25 °C). The probes were subjected to electrophoresis in the 4% polyacrylamide gel (acrylamide: bisacrylamide 59:1; 0.2× TBE buffer) at 120 V for 90 min. Ladder GeneRuler 100 bp (Thermo Fisher Scientific, Waltham, MA, USA) was used as a marker.

The fluorescent imaging of the gels was performed using the Amersham Typhoon RGB imager (GE Healthcare Bio Sciences AB, Uppsala, Sweden).

### 4.8. Western Blotting

WB was carried out using the protocols published earlier [35,49]. Briefly, the probes (20 µL) for WB were prepared as for the electrophoresis (see above), mixed with 5 µL of the 5× loading buffer (312 mM Tris-HCl pH 6.8, 10% SDS, 25% β-ME, 0.05% bromophenol blue) and then heated to 95 °C for 5–10 min. After that, the probes were subjected to electrophoresis in the 4–12% bis-Tris gradient gel in the Tris-glycine/SDS running buffer (25 mM Tris-HCl, 192 mM glycine and 0.1% SDS, pH 8.6) at 130 V for 1 h at RT. The protein transfer on the supported nitrocellulose membrane (Bio-Rad) was performed in the transfer buffer (50 mM MOPS, 50 mM Tris-HCl, 1 mM EDTA, 3.5 mM SDS) with 20% ethanol at 4 °C (350 mA, 2 h). The membrane was incubated for 60 min in the PBS-T solution (2.7 mM KCl, 8 mM Na_2_HPO_4_, 2 mM KH_2_PO_4_, 37 mM NaCl, 0.5% Tween 20) supplemented with 5% skimmed milk (prepared from dry powder) and washed twice with the 1× PBS-T solution for 5 min. Then, the membrane was incubated with mouse monoclonal antibodies against PAR (catalog number ab14459, Abcam, Waltham, MA, USA) for 1 h in PBS-T/5% milk, washed, incubated with the secondary antimouse antibodies conjugated with horseradish peroxidase (Bio-Rad, Hercules, CA, USA) for 1 h in the PBS-T/5% milk solution and washed again. Immunodetection was performed using the Chemidoc reader (Bio-Rad) and the Super Signal West Pico chemiluminescent substrate (Thermo Fisher Scientific, Waltham, MA, USA) for 3 min.

### 4.9. Statistical Analysis

All the presented results were reproduced in at least three independent experiments. The frequency distributions of nucleosomes in the E_PR_ value (E_PR_ profiles) were averaged (mean ± SEM, *n* = 3, 3000–16,000 particles) over three independent experiments. To determine the significance of the revealed differences, an unpaired two-tailed t-test was used. The difference between the values was considered as significant if *p* < 0.05.

## 5. Conclusions

Natural polyphenols are actively studied as potential drugs or as companions of existing drugs for the treatment of human diseases. Studies of the mechanisms of the action of polyphenols intend to reveal their pharmacological targets and adverse effects, and to define in this way possible clinical applications. EGCG is one of the polyphenols possessing a wide range of well-established health promoting effects [1,2,3,4]. Some mechanisms of EGCG action and its molecular targets have already been identified [9,11,12,13,14,15,16,19]. The aim of our study was to fill the existing gap in understanding the effect of EGCG on chromatin. The findings of our study could be summarized as follows:

- EGCG very effectively penetrated into the cell nuclei and bound to DNA there.
- EGCG induced a large-scale uncoiling of nucleosomal DNA from the histone octamer; this process was reversible and was not accompanied by the dissociation of core histones from DNA.
- EGCG reorganized the structure of chromatosomes, very likely without the dissociation of the linker histone.
- EGCG did not reduce the affinity of PARP1 to nucleosomes.
- EGCG affected the structure of the PARP1–nucleosome complex.
- The auto-PARylation activity of PARP1 did not change in the presence of sub- and low-micromolar concentrations of EGCG.

Since interactions of many others polyphenols with chromatin also need to be investigated, our experimental approach based on the combination of fluorescence microscopy of living cells and spFRET microscopy of nucleosomes could be applied for the study of these polyphenols.

## Data Availability

The data presented in this study are available on request from the corresponding authors. The data are not publicly available due to local regulations.

## Supplementary Materials

The supporting information can be downloaded at: https://www.mdpi.com/article/10.3390/ijms241814187/s1.
