# Supplementary material for: Epigallocatechin Gallate Affects the Structure of Chromatosomes, Nucleosomes and Their Complexes with PARP1
Source: Int J Mol Sci. 2023 Sep 16;24(18):14187. doi: 10.3390/ijms241814187 (PMC10532227; doi:10.3390/ijms241814187)
Supplement: Supplementary file 1 [file ijms-24-14187-s001.zip › ijms-2601878-supplementary.pdf]

Supplementary materials to

Epigallocatechin gallate affects the structure of chromatosomes, nucleosomes and their complexes with PARP1

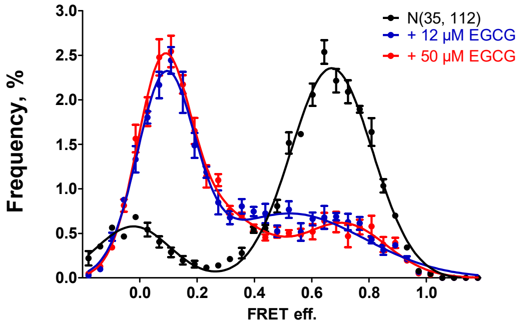

Figure S1. Frequency distributions of nucleosomes N(35, 112) by E_{PR} value in the absence and presence of 12 and 50 μM EGCG (mean±SEM, n=3).

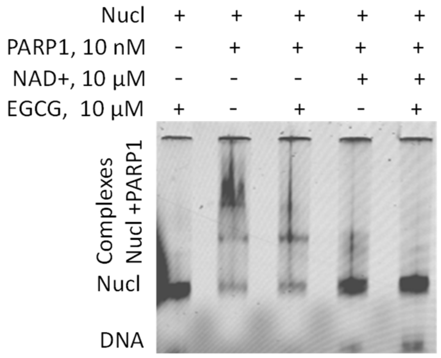

Figure S2. Analysis of the complexes of nucleosomes with PARP1, EGCG and NAD+ by non-denaturing PAGE.

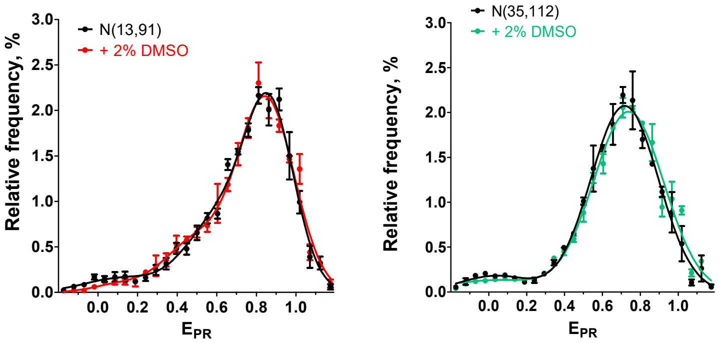

Figure S3. Frequency distributions of nucleosomes by E_{PR} value in the absence and presence of 2% DMSO (mean±SEM, n=3).
